# A bibliometric analysis of biophilic architectural principles: global trends and research evolution

**DOI:** 10.3389/frma.2026.1914242

**Published:** 2026-08-04

**Authors:** Eghosa Noel Ekhaese, David Oluwagbenga Adegbola

**Affiliations:** Department of Architecture, College of Science and Technology, Covenant University, Canaanland, Nigeria

**Keywords:** architecture, bibliometric analysis, biophilic design, built environment, research trends

## Abstract

Biophilic architectural design has evolved from a niche philosophy into a mainstream strategy for public health and climate resilience. This study presents a bibliometric analysis of the global biophilic research landscape over a 20-year horizon (2006–2026). Using VOSviewer, a fractional counting network analysis was conducted on 66 high-frequency author keywords from the Scopus database. The intellectual structure centers on four distinct thematic clusters: macro-urban climate resilience (Theme A); indoor environmental quality and building performance physics (Theme B); environmental psychology and restorative workspaces (Theme C); and typological sector deployments linked to workplace productivity (Theme D). Timeline overlays reveal a shift from foundational, conceptual work (2006–2016) to technical engineering integration (2017–2021), and more recently to a focus on resilience, digital simulation, and post-pandemic wellness architecture (2022–2026). Geographic mapping shows that publication output remains heavily concentrated in Global North economies, with comparatively limited representation from the Global South, a pattern that may reflect, among other factors, differences in research funding, database indexing, and language coverage rather than differences in the relevance or need for biophilic strategies in those regions. These findings offer a data-informed starting point for practitioners and researchers seeking to broaden biophilic design's evidence base across climatic, economic, and geographic contexts, and to strengthen benchmarks for socio-economic equity and long-term post-occupancy performance.

## 1 Introduction

Biophilic design has attracted growing interest in recent years, largely because of its potential to improve human health, wellbeing, and sustainability within the built environment (Hähn et al., 2021; Zhong et al., 2022). At its core, this design philosophy seeks to reconnect people with nature by weaving natural elements, materials, and processes into the spaces where we live and work.

Biophilia is the inherent human inclination to connect with nature and other living systems (Jude et al., 2025; Sultan Qurraie and Bayram, 2025; Aranda Mena, 2018). First defined by Fromm (1964) and Wilson (1984), this concept suggests that humans have a genetic need to affiliate with the natural world for physical and mental health (Aabouelela, 2023; Tekin et al., 2023; Alam, 2023). In contemporary society, built structures have largely replaced natural habitats, as people spend approximately 90% of their time within indoor environments (Elantary, 2024; Tabassum and Park, 2024; Radha, 2022), and this spatial isolation from nature is often linked to sensory deprivation and chronic stress (Kavathekar and Bantanur, 2021; Asojo and Hazazi, 2025).

The case for biophilic design begins with a well-documented body of evidence linking exposure to nature with better health outcomes (Adekanmbi et al., 2024). Access to natural light and views of the outdoors, for instance, have been shown to lower stress levels, lift mood, and boost productivity, outcomes that matter deeply in both residential and workplace settings. This sense of connection to the natural world carries particular significance in urban contexts, where nature is often scarce.

Attempts to categorize and standardize biophilic design have produced several notable frameworks. Cramer and Browning (2008) proposed three broad categories for understanding human and nature relationships: nature in space, natural analogs, and the nature of the space. Kellert et al. (2008) similarly identified six biophilic elements, later formalized by Browning et al. (2014) into fourteen design patterns organized under the same three categories. Kellert and Calabrese (2015) offered a more detailed taxonomy, identifying twenty four biophilic criteria across three categories, while Browning and Ryan (2020) refined this further into fifteen criteria, also organized under three headings. A comparative overview of these classification systems is presented in Table 1.

**Table 1:** Biophilic design frameworks.

Kellert et al. (2008) biophilic design patterns					
Environmental Features	Natural shapes and forms	Natural patterns and processes	Light and space	Place-based relationships	Evolved Human-Nature Relationship
✓ Color ✓ Water ✓ Air ✓ Sunlight ✓ Plants ✓ Animals ✓ Natural material ✓ Views and vistas Façade greening ✓ Geology and landscape ✓ Habitats and ecosystems ✓ Fire	✓ Botanical motifs ✓ Tree and columnar supports ✓ Animal (mainly vertebrate) motifs ✓ Shelles and tubular forms ✓ Arches, vaults, domes ✓ Shapes resisting straight lines and right angles ✓ Simulation of natural features ✓ Biomorphy ✓ Geomorphology ✓ Biomimicry	✓ Sensory variability ✓ Information richness Age, ✓ change, and the patina of time ✓ Growth and efflorescence ✓ Central focal point ✓ Patterned wholes ✓ Bounded spaces ✓ Transitional spaces ✓ Linked series and chains ✓ Integration of parts into wholes ✓ Complementary contrasts ✓ Dynamic balance and tension ✓ Fractals ✓ Hierarchically organised ratios and scales	✓ Natural light ✓ Filtered and ✓ diffused light ✓ Light and shadow ✓ Reflected light ✓ Lightpools ✓ Warm light ✓ Light as shape and form, Spaciousness ✓ Spatial variability ✓ Space as shape and form: Spatial harmony ✓ Inside-outside spaces	✓ Geographic connection to the place ✓ Historic connection to place ✓ Ecological connection to place ✓ Cultural connection to place ✓ Indigenousmaterials ✓ Landscape orientation ✓ Landscape features that define a building ✓ Landscape ecology ✓ Integration of culture and ecology ✓ Spirit of place ✓ Avoiding placelessness	✓ Prospect and refuge ✓ Order and complexity ✓ Curiosity and enticement ✓ Change and metamorphosis ✓ Security and protection ✓ Mastery and control ✓ Affection and attachment ✓ Attraction and beauty ✓ Exploration and discovery ✓ Information and cognition ✓ Fear and awe, reverence and spirituality
**Kellert (** **2018** **) biophilic design patterns**					
**Direct Experience of Nature**		**Indirect Experience of Nature**		**Experience of Space and Place**	
Light, air, water, plants, animals, landscapes, weather, views and fire		Images, Materials, Texture, Colour, Shapes and forms, Information richness, Change, age and the patina of time, Natural geometries, Simulated natural light and air, Biomimicry		Prospect and refuge, Organised complexity, Mobility, Transitional spaces, place, Integrating parts to create wholes	
**Browning and Ryan (** **2020** **) biophilic design patterns**					
**Nature in the Space**		**Natural Analogues**		**Nature of the Space**	
✓ Visual Connection with Nature ✓ Non-Visual Connection with Nature Non-Rhythmic Sensory Stimuli ✓ Thermal & airflow variability ✓ Presence of water ✓ Dynamic & diffuse light ✓ Connection with natural systems		✓ Biomorphic forms & patterns ✓ Material connection with nature ✓ Complexity & order		✓ Prospect ✓ Refuge ✓ Mystery ✓ Risk/peril Awe	

Source: Authors' survey, 2026.

These three frameworks serve a shared purpose: to help designers understand and apply the principles of biophilic design in practice (Zhong et al., 2022), providing structured guidance for incorporating biophilic criteria into the built environment (Abo Sabaa et al., 2022; Wijesooriya et al., 2023; Tabassum and Park, 2024).

Several reviews have already sought to consolidate this literature, though each stops short of a large scale, quantitative mapping of the field. Zhong et al. (2022) offer a critical review of biophilic design's contribution to health and sustainability, but their synthesis is narrative and conceptual rather than data-driven, and does not quantify publication trends, influential contributors, or thematic structure across the field. Wijesooriya et al. (2023) review existing biophilic design frameworks and their compatibility with sustainability certification systems, but their focus is limited to comparing named frameworks rather than mapping the wider research landscape. Tekin et al. (2023) conduct a systematic review of biophilic design parameters, but restrict their scope to clinical environments alone. Sultan Qurraie and Bayram (2025) provide a research review of biophilic design's impact, but similarly rely on narrative synthesis rather than bibliometric mapping. What remains missing is a quantitative, data-driven account of how the field as a whole has developed: which themes have emerged and receded, which authors, institutions, and countries have driven that development, and how these patterns have shifted over a substantial time horizon.

The application of biophilic strategies has proven effective across diverse building typologies. In healthcare, biophilic stimuli accelerate patient recovery and reduce burnout among staff (Arriazu Ramos et al., 2024; Eja et al., 2025; Maurya et al., 2024). Educational facilities utilize natural light and greenery to enhance student concentration and academic performance (Ghaziani et al., 2021; Azizah et al., 2025; Chabok et al., 2025; Fadda et al., 2023). In workplaces, biophilic interventions boost employee productivity and job satisfaction (Permana et al., 2020; Tharim et al., 2023; Li et al., 2025). Residential applications show a significant positive correlation with mood improvement and overall wellbeing (Obeidat and Obeidat, 2025; Maharani and Fitriyanto, 2022; Priyanto, 2025; Bulaj et al., 2025), while public spaces and hospitality settings also benefit from these principles, enhancing guest satisfaction and community cohesion (Ariyawansa and Perera, 2022; Ariyani et al., 2022).

Beyond human centric benefits, biophilic architecture is increasingly recognized for its contribution to sustainable design goals, including improved energy efficiency and indoor environmental quality (Ailyn, 2025; Agboola, 2024; Deshpande et al., 2024; Asojo and Hazazi, 2025). The COVID-19 pandemic served as a major catalyst for a surge in research, as lockdowns highlighted the critical importance of nature-integrated indoor spaces for public health and urban resilience (Bahador and Mahmoudi Zarandi, 2023; Sultan Qurraie and Bayram, 2025). Despite this prolific growth, current literature remains fragmented across specific typologies and geographical regions (Sultan Qurraie and Bayram, 2025).

This study responds to that need by offering a data-driven understanding of the field to guide both future research and design practice. To that end, this study examines global research on biophilic architectural design published between 2006 and 2026, with a focus on bibliometric indicators including publication trends, citation patterns, authorship, geographic distribution, and keyword analysis. The scope is limited to data drawn from a selected academic database and does not extend to a detailed content analysis of individual papers. The overarching aim is to map the global research landscape of biophilic architectural design, tracing the evolution of key themes and identifying knowledge gaps that can inform the direction of future inquiry. In pursuit of this aim, the study addresses the following research questions:

i. What are the global publication trends in biophilic architectural design research between 2006 and 2026?
ii. Who are the leading authors, institutions, countries, and journals contributing to the field of biophilic architectural design?
iii. What are the dominant thematic clusters, semantic relationships, and chronological keyword evolutions that define the intellectual framework of biophilic architecture?
iv. What critical knowledge gaps persist within the existing literature, and what strategic directions should govern future research frameworks in biophilic design?

## 2 Materials and methods

This study adopted a quantitative bibliometric approach to examine global research trends in biophilic architecture and design. The methodology was designed to ensure transparency, consistency, and reliability across both data collection and analysis. The study was carried out in two main stages. The first stage focused on retrieving relevant publications from a reliable academic database, while the second involved analyzing and visualizing the retrieved data to identify major research trends, themes, and scholarly contributions.

### 2.1 Research design and data source selection

Bibliometric analysis was selected as the primary methodology owing to its effectiveness in examining large volumes of scientific literature through objective, data-driven methods. Elsevier's Scopus database was chosen as the main data source, given its broad coverage of peer-reviewed journals spanning architecture, environmental studies, engineering, and related disciplines. Scopus was further preferred for its robust citation indexing and its established reliability in large scale academic analysis.

### 2.2 Search strategy and bibliographic query

The search was run on May 28, 2026. This date is reported because Scopus is updated continuously, and defines the retrieval snapshot underlying the reported counts. The search targeted keywords in titles, abstracts, and author keywords (TITLE ABS KEY), combined with Boolean operators. The full query string used was:

TITLE ABS KEY (“biophilic design” OR “biophilic architecture” OR “biophilic principles” OR “human nature design”) AND (LIMIT TO (DOCTYPE, “ar”) OR LIMIT TO (DOCTYPE, “re”)) AND (LIMIT TO (LANGUAGE, “English”)) AND (PUBYEAR > 2006 AND PUBYEAR < 2026).

The initial search returned 1,000 documents, refined to 641 (574 journal articles, 67 review papers).

**Figure 1:**
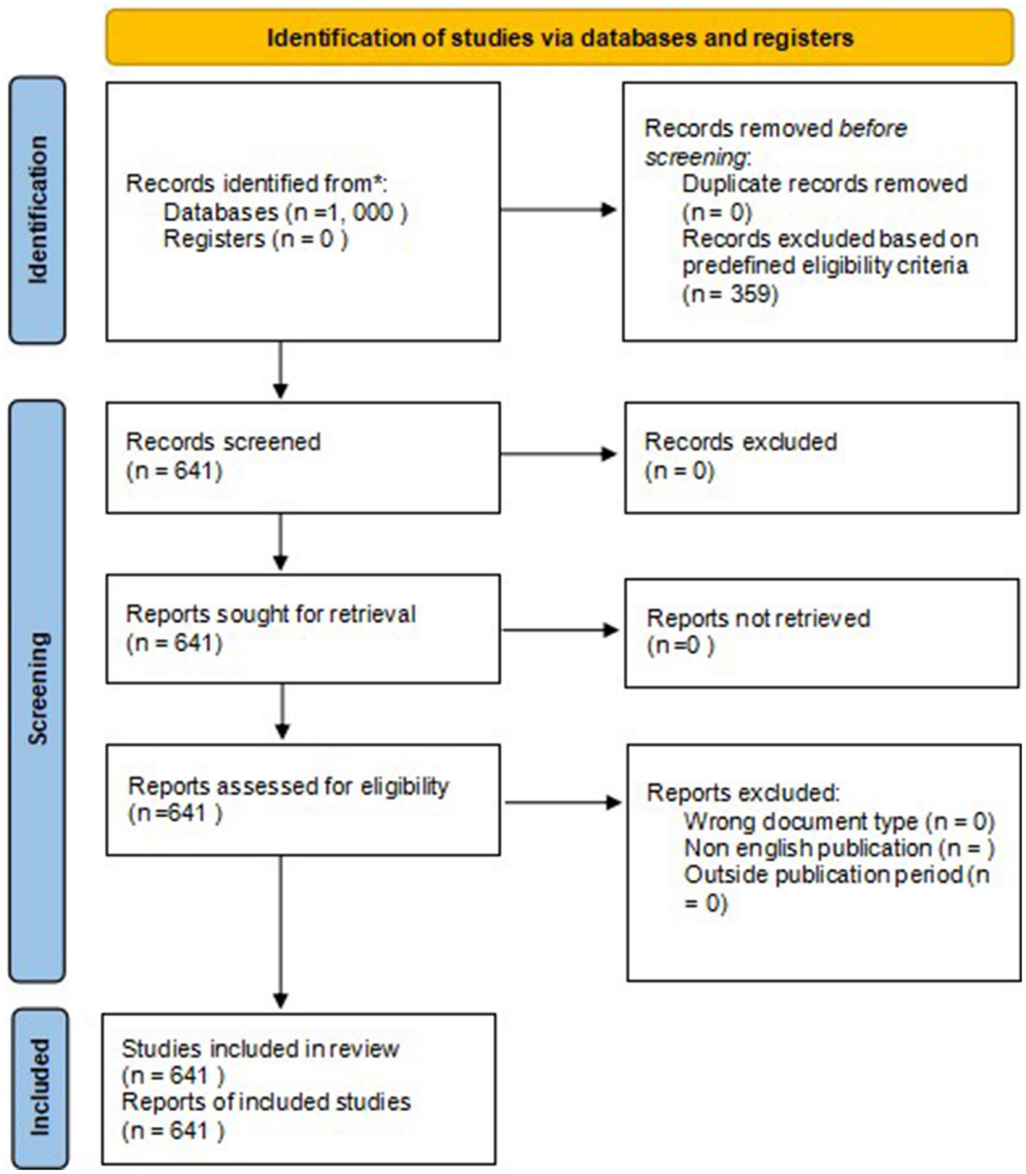
PRISMA-style flow diagram of the identification, screening, and inclusion process. Source: Adapted from Page et al. (2021).

As shown in Table 2 and Figure 1, the initial search returned 1,000 documents. Following the application of selection criteria, the dataset was refined to 641 publications, comprising 574 journal articles and 67 review papers. Other document types, including conference papers, editorials, book chapters, and trade publications, were excluded to maintain data quality and consistency. The final dataset spans publications from 2006 to 2026.

**Table 2:** Scopus database search criteria and query string.

Database Source	Search Field / Field Tag	Search for Query String Syntax	Initial Hits	Refined Dataset
Scopus	TITLE-ABS-KEY	Full string above	1,000	641

Source: Authors Research Design (2026).

### 2.3 Inclusion and exclusion criteria

To safeguard the relevance and quality of the dataset, clear inclusion and exclusion criteria were applied from the outset. Only publications written in English were considered. Within that scope, the study included only peer-reviewed journal articles and review papers, while conference proceedings, editorials, notes, and book reviews were excluded. A publication window spanning 2006 to 2026 was adopted to capture the development of biophilic architecture from its early theoretical foundations through to more recent discussions of sustainability, wellbeing, and climate responsive design. Documents that did not satisfy all three structural parameters were filtered out using the automated sidebar configuration controls available within the database.

### 2.4 Data extraction and analytical software pipeline

After filtering, the bibliographic records were exported from Scopus in CSV, including author names, affiliations, article titles, abstracts, journals, citation counts, and keywords. The data were analyzed using VOSviewer (Version 1.6.20). VOSviewer was used for keyword co-occurrence network mapping. The network was constructed using fractional counting (rather than full counting), which weights each co-occurrence by the number of keywords in the document, preventing multi-keyword papers from disproportionately inflating link strength. Clustering was performed using VOSviewer's default resolution parameter of 1.00, with a minimum keyword occurrence threshold of five as noted above. These parameters produced the four thematic clusters (Themes A–D) reported in Section 3.

The same fractional counting method and resolution parameter of 1.00 were also applied to construct co-authorship networks (at both author and country level) and co-citation networks of cited references, using the full bibliographic export rather than the keyword subset. These additional analyses are reported in Section 3.2.4.

## 3 Results and discussions

Following the filtering process, bibliographic records were exported from Scopus, encompassing author names, affiliations, article titles, abstracts, journals, citation counts, and keywords. The exported data were then analyzed using two tools: Microsoft Excel and VOSviewer (Version 1.6.20). Microsoft Excel was used to conduct descriptive statistical analysis, covering publication trends, leading countries, contributing institutions, and journal outputs. VOSviewer was employed for science mapping and keyword co-occurrence analysis, helping to visualize relationships between frequently appearing keywords and to identify major thematic clusters within the field. The resulting network maps revealed key research areas and illuminated how the focus of biophilic architecture research has shifted over time. Taken together, this analytical process offered a clearer picture of the field's intellectual structure and the trajectory of its development.

### 3.1 RQ1: What are the global publication trends in biophilic architectural design research between 2006 and 2026?

**Figure 2:**
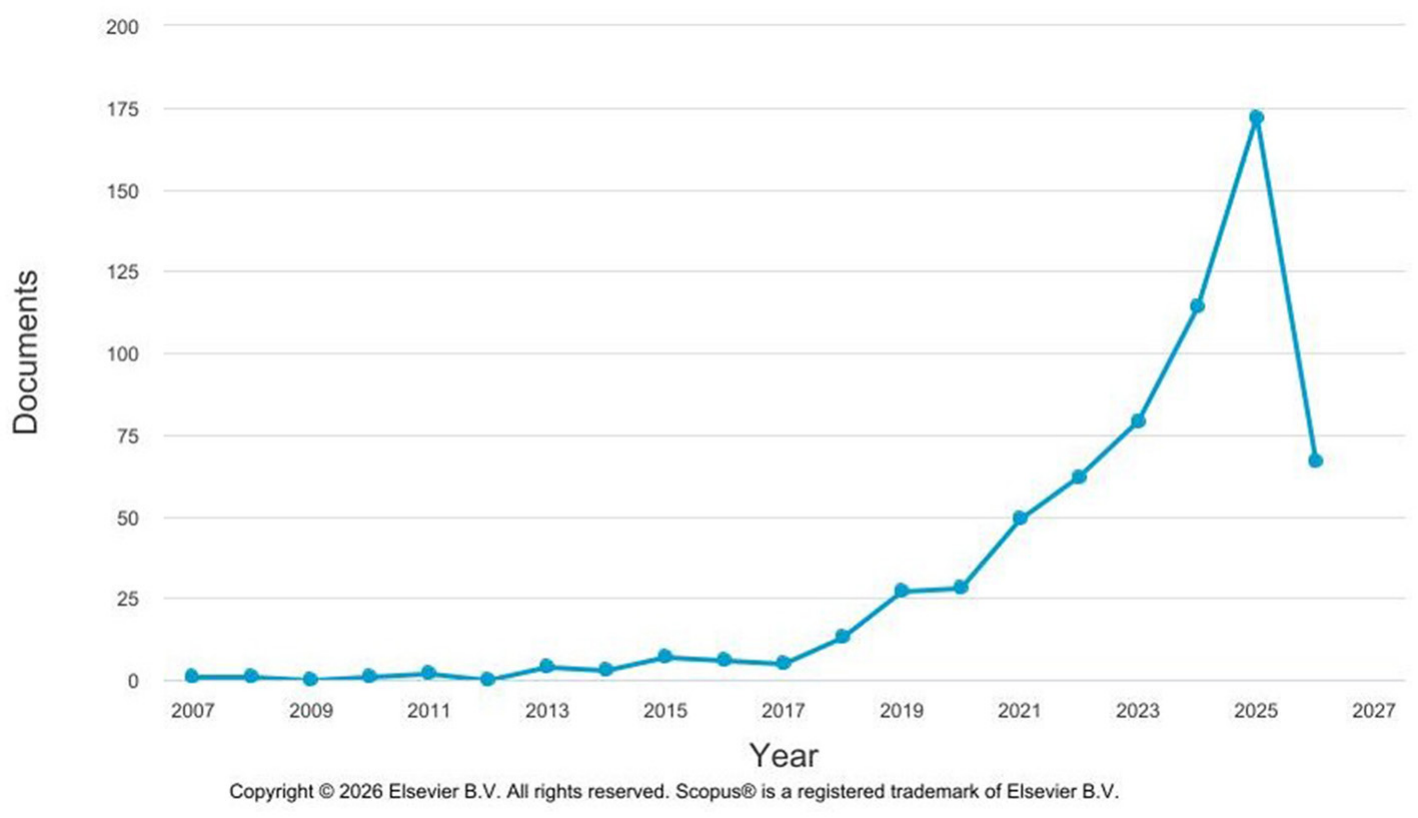
Chronological trajectory of annual scientific publications and cumulative citation impacts (2006–2026); Source: Scopus / Authors Analysis (2026).

The chronological distribution of peer-reviewed publications retrieved from the Scopus database reveals a distinct three stage evolutionary trajectory spanning the last two decades. As shown in Figure 2, the first phase, described here as the incubation stage (2006 to 2017), was characterized by relatively low research output, with fewer than ten publications produced annually. During this period, studies on biophilia remained largely theoretical in nature, closely tied to environmental psychology and human and nature relationships, with limited uptake in mainstream architectural practice.

A gradual but noticeable increase in research output began to emerge between 2018 and 2021 (Figure 2). Publication volumes rose from 13 studies in 2018 to approximately 50 by 2021, a growth that reflects the broadening recognition of biophilic principles across sustainable design, indoor environmental quality, and human centered architecture.

As shown in Figure 2, the most significant expansion occurred between 2022 and 2025, when the field experienced a sharp rise in academic interest. Publications peaked in 2025, with 172 studies recorded. This rapid growth may be attributed to the heightened global attention directed at health conscious spaces, wellness oriented environments, and climate responsive design in the aftermath of the COVID-19 pandemic. The overall trend suggests that biophilic design has firmly established itself as an important area of inquiry within contemporary built environment research.

Although the publication count appears lower for 2026 (*n* = 67), this does not necessarily signal a decline in scholarly interest. Rather, it reflects the fact that the indexing year is still in progress and that the database remains incomplete at the point of data collection.

### 3.2 RQ2: Who are the leading authors, institutions, countries, and journals contributing to the field of biophilic architectural design?

#### 3.2.1 Prolific Authors and Core Citation Patterns

**Figure 3:**
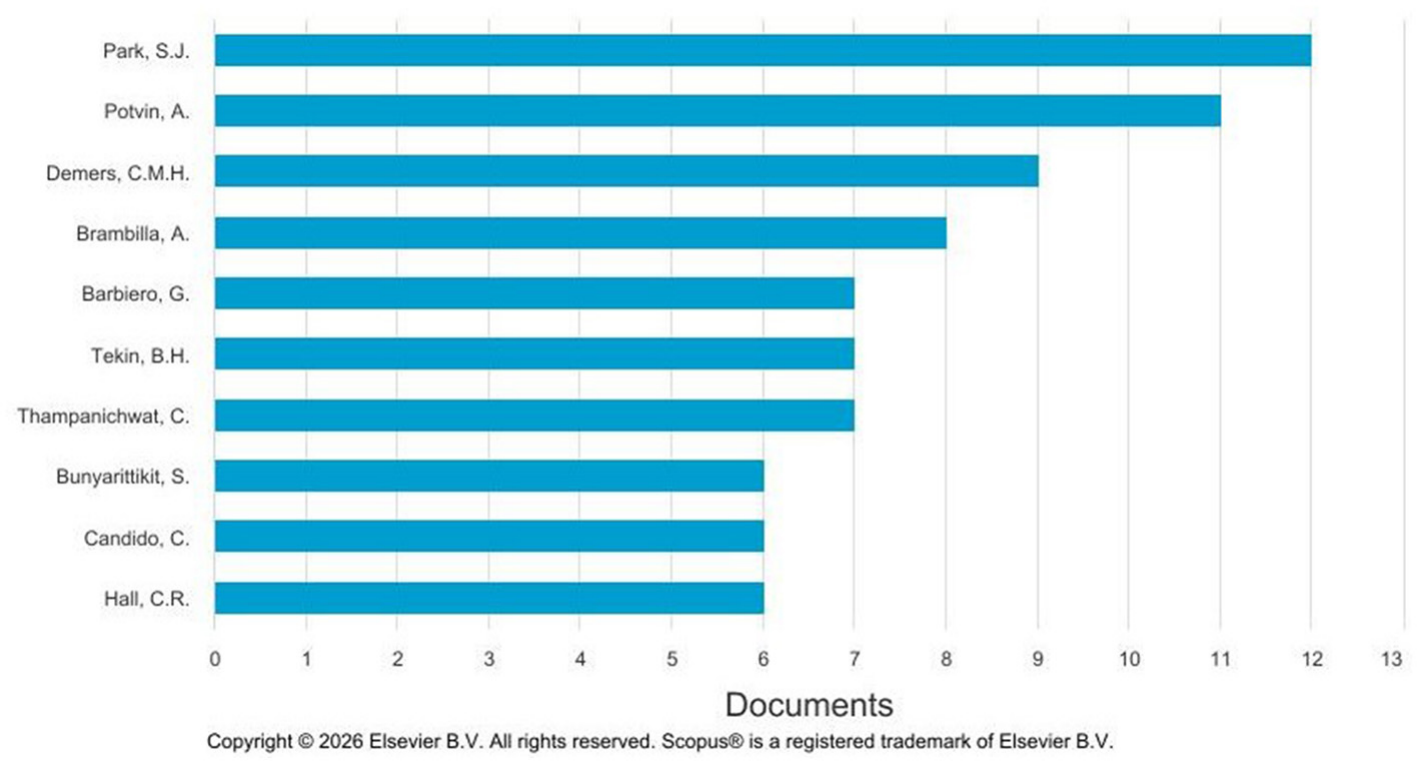
Comparative analysis of the top 10 most productive authors by publication volume within biophilic architectural discourse. Source: Scopus / Authors Analysis (2026).

Intellectual leadership within the field is broadly distributed, though distinct scholarly clusters emerge among the most prolific contributors (Table 3). As shown in Figure 3, Park, S.J. leads in terms of output with 12 indexed documents, followed closely by Potvin, A. (*n* = 11) and Demers, C.M.H. (*n* = 9). The prominent positioning of Potvin and Demers points to strong collaborative research networks centered on passive daylighting, microclimates, and experiential natural patterns within institutional settings. The foundational contributions of Brambilla, A. (*n* = 8), Barbiero, G. (*n* = 7), and Candido, C. (*n* = 6) further reflect a well established research stream dedicated to validating the empirical, biological, and physiological effects of biophilic interventions on building occupants.

**Table 3:** Leading authors by number of publications and their percentage contribution to the total 641 articles included in the bibliometric analysis.

S/N	Author	Number of Publications	Percentage of Total (641)
1	Park, S.J.	12	1.72%
2	Potvin, A.	11	1.56%
3	Demers, C.M.H.	9	1.40%
4	Brambilla, A.	8	1.25%
5	Barbiero, G.	7	1.09%
6	Tekin, B.H.	7	1.09%
7	Thampanichwat, C.	7	1.09%
8	Bunyarititkit, S.	6	0.94%
9	Candido, C.	6	0.94%
10	Hall, C.R.	6	0.94%

#### 3.2.2 Most productive journals and source dynamics

**Figure 4:**
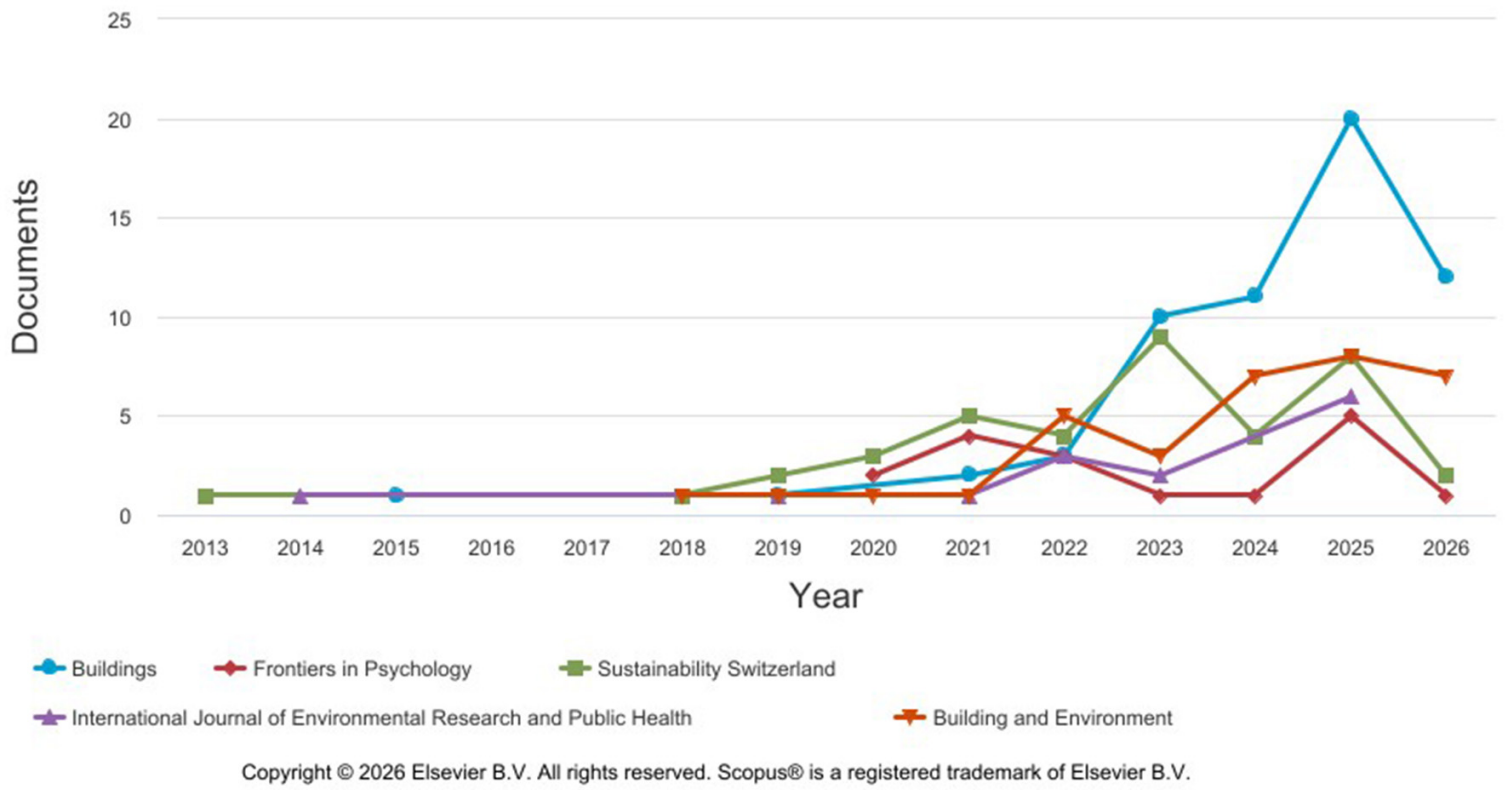
Chronological publishing trajectories of the top 5 core academic journals specialising in biophilic architecture (2013–2026). Source: Scopus / Authors Analysis (2026).

As shown in Table 4, an analysis of publishing channels reveals that the dissemination of biophilic research is heavily concentrated in high-impact, peer-reviewed journals that bridge architectural science and environmental sustainability (Figure 4).

**Table 4:** Core academic journal outlets in biophilic architecture.

Journal source	General trend & role in the field
Buildings	Establishes absolute dominance in production volume, experiencing an exponential spike from 2022 onward and peaking at 20 publications in 2025. This profile positions it as the premier outlet for structural and engineering applications of biophilia.
Building and Environment	Exhibits a highly stable, upward trajectory, maintaining consistent growth through 2024–2025 (n=8). It serves as the primary repository for empirical assessments of indoor microclimates and environmental physics.
Sustainability (Switzerland)	Served as an early pioneer, experiencing a notable publication peak in 2023 (n=9) before seeing a more decentralised distribution of articles in subsequent years.
Frontiers in Psychology & IJERPH	Both maintain a continuous, steady presence. The steady performance highlights that the biophilic discourse remains deeply rooted in interdisciplinary public health and psychological restoration frameworks.

Source: Scopus / Authors Analysis (2026).

#### 3.2.3 Leading countries, regional trends, and key affiliated institutions

The geographical distribution of publications reveals that research on biophilic design remains concentrated in a small number of major economies, though contributions are increasingly spread across a wider range of regions. The United States recorded the highest output (*n* = 119), establishing it as the leading contributor to the field. This strong performance may be linked to the country's early role in developing biophilic design theories, alongside continued investment in sustainable and healthy building practices. China (*n* = 63) and Australia (*n* = 61) followed as the next most significant contributors. China's growing research presence likely reflects rising attention to sustainable urban development and green city initiatives, while Australia's output points to sustained interest in healthy buildings, workplace wellbeing, and environmentally responsive design.

**Figure 5:**
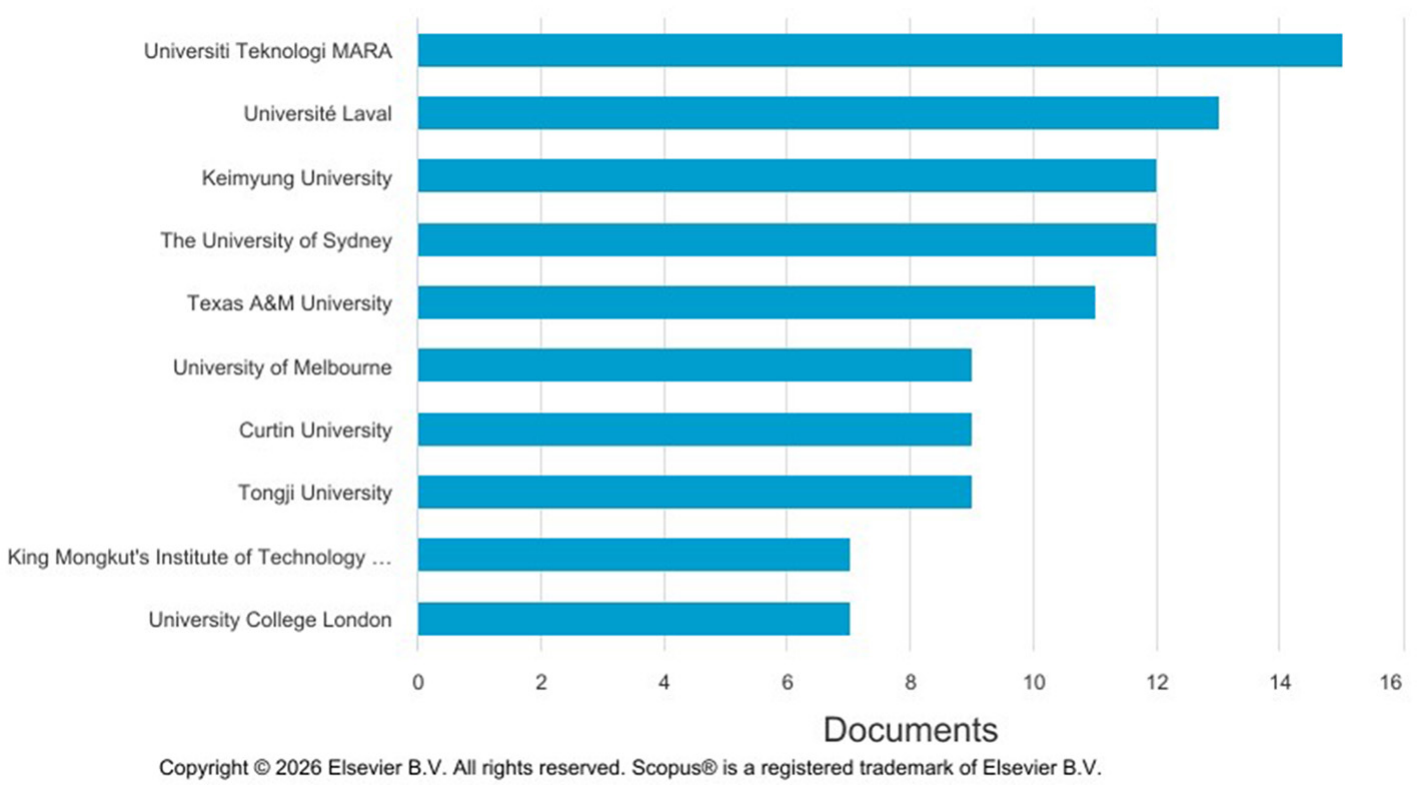
Institutional ranking of the top 10 global academic affiliations driving biophilic spatial research. Source: Scopus / Authors Analysis (2026).

While certain countries dominate in overall output, a closer look at institutional contributions reveals a more globally distributed research landscape. Universiti Teknologi MARA recorded the highest institutional output with 15 publications (Figure 5), suggesting growing scholarly attention to biophilic design in tropical and hot-humid climate contexts. Université Laval followed with 13 publications, while Keimyung University and the University of Sydney each contributed 12 publications, reflecting strong research activity across the Asia Pacific region.

Texas A&M University (*n* = 11) and Tongji University (*n* = 9) also emerged as notable contributors (Figure 5). Taken together, these findings suggest that while research leadership remains concentrated in a handful of countries, institutional productivity is more evenly distributed, pointing to a broadening base of international interest in biophilic architecture and sustainable built environments.

#### 3.2.4 Citation analysis: most influential publications

Citation analysis was conducted using the Cited by counts available in the Scopus database to identify the most influential publications within the dataset. The results indicate that a relatively small number of publications account for a substantial proportion of the field's scholarly influence, reflecting the foundational role of these studies in shaping contemporary biophilic architectural research.

As shown in Table 5, the most cited publication was Yin et al. (2020), Effects of biophilic indoor environment on stress and anxiety recovery: A between-subjects experiment in virtual reality, with 358 citations. This was followed by Yin et al. (2018) with 346 citations, and Zhong et al. (2022) with 322 citations. Other highly cited studies include Gillis and Gatersleben (2015), Ryan et al. (2014), and Beatley and Newman (2013), all of which have made significant contributions to understanding the theoretical foundations, health benefits, and urban applications of biophilic design. Overall, the citation distribution demonstrates that the field is anchored by a combination of foundational theoretical works, empirical investigations, and comprehensive review articles that continue to shape subsequent research.

**Table 5:** Top 10 most cited publications.

Rank	First author	Year	Citations	Short title
1	Yin et al.	2020	358	Effects of biophilic indoor environment on stress and anxiety recovery
2	Yin et al.	2018	346	Physiological and cognitive performance of exposure to biophilic indoor environment
3	Zhong et al.	2022	322	Biophilic design in architecture and its contributions to health, wellbeing, and sustainability
4	Gillis & Gatersleben	2015	306	Health and wellbeing benefits of biophilic design
5	Ryan et al.	2014	305	Biophilic design patterns
6	Ko et al.	2020	292	Window views, thermal comfort, emotion, and cognitive performance
7	Beatley & Newman	2013	260	Biophilic cities are sustainable, resilient cities
8	Yin et al.	2019	202	Biophilic interventions in offices
9	Yeom et al.	2021	149	Psychological and physiological effects of a green wall
10	Gaekwad et al.	2022	136	Meta-analysis of emotional evidence for the biophilia hypothesis

Source: Authors Analysis (2026).

#### 3.2.5 Co-authorship analysis: international collaboration patterns

**Figure 6:**
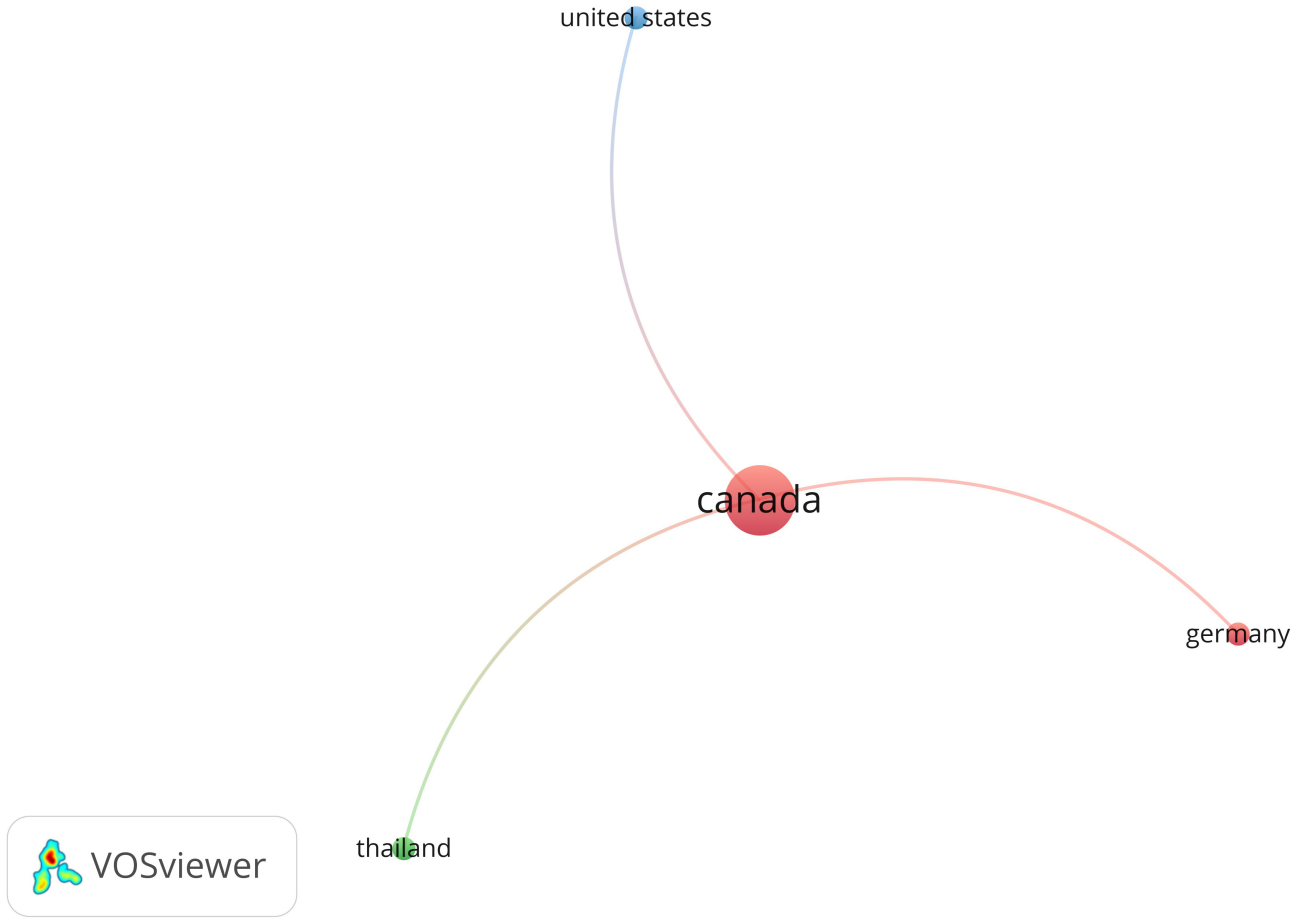
Co-authorship network of countries contributing to biophilic architectural design research (n = 4 connected countries). VOSviewer / Authors Analysis (2026).

A country-level co-authorship analysis was conducted using VOSviewer with fractional counting and a minimum threshold of one document per country. Of the 12 countries meeting this threshold, only four, Canada, the United States, Germany, and Thailand, formed a connected co-authorship network, with Canada occupying the central position linking the other three (Figure 6).

This result stands in notable contrast to the publication volume findings reported in Section 3.2.3, where the United States, China, and Australia emerged as the leading contributors by output. Despite their high individual publication counts, these countries do not appear as prominent nodes within the international collaboration network, indicating that their research output is driven predominantly by domestic authorship teams rather than cross-border partnerships. Canada's central position, despite not leading in raw publication volume, suggests that Canadian institutions play a disproportionately connective role in whatever limited international collaboration does occur within the field.

Overall, this finding indicates that international collaboration in biophilic architecture research remains limited in both scale and reach. Rather than forming a broadly interconnected global research community, the field appears to be characterized by largely independent national research clusters, with only sparse cross-border linkages. This is consistent with the geographic fragmentation identified through the keyword and thematic analysis in Sections 3.3 and 3.4.1, and reinforces the broader observation that biophilic design knowledge production, despite being geographically widespread, is not yet matched by an equally distributed pattern of international scholarly collaboration.

#### 3.2.6 Co-citation analysis: intellectual structure of the field

**Figure 7:**
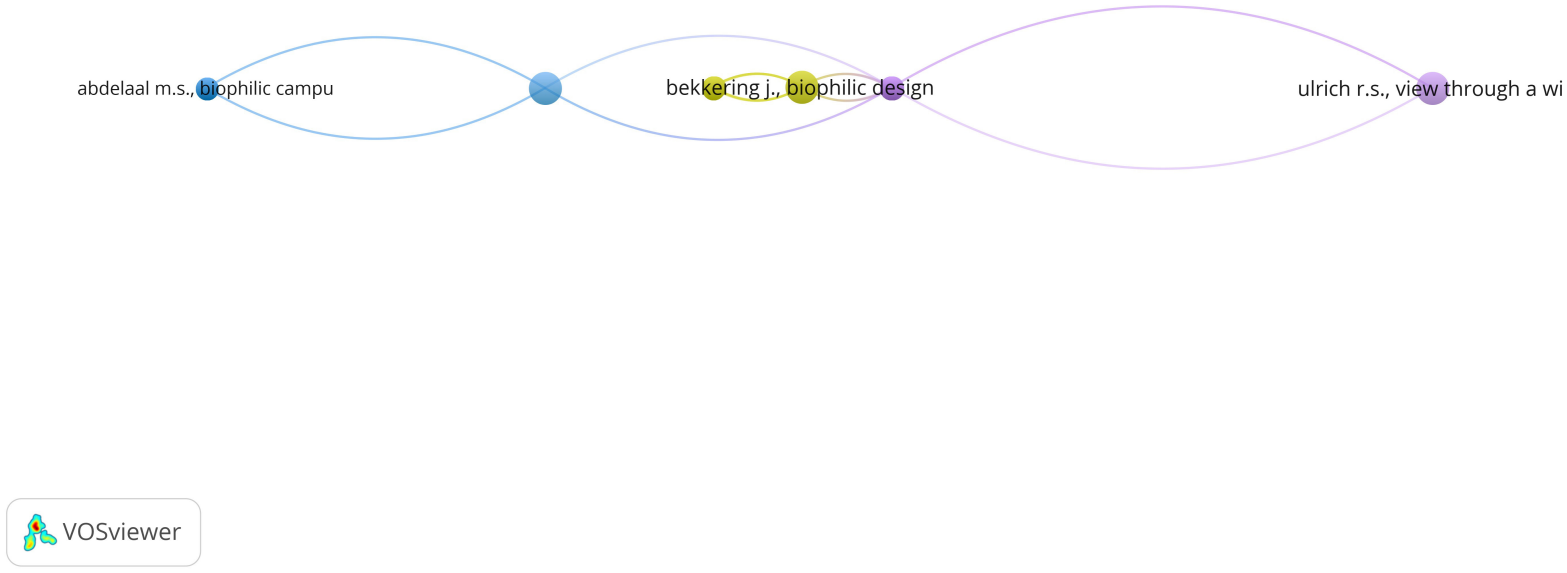
Overview of the co-citation network showing core bridging references across thematic clusters. Source: VOSviewer / Authors Analysis (2026).

To examine the intellectual foundations underlying biophilic architecture research, a co-citation analysis was conducted on the cited references within the 641-document dataset, using VOSviewer with fractional counting and a minimum threshold of 2 citations per reference. Of the 531 cited references meeting this threshold, 381 formed a single connected network, organized into three distinct thematic clusters (Figure 7).

**Figure 8:**
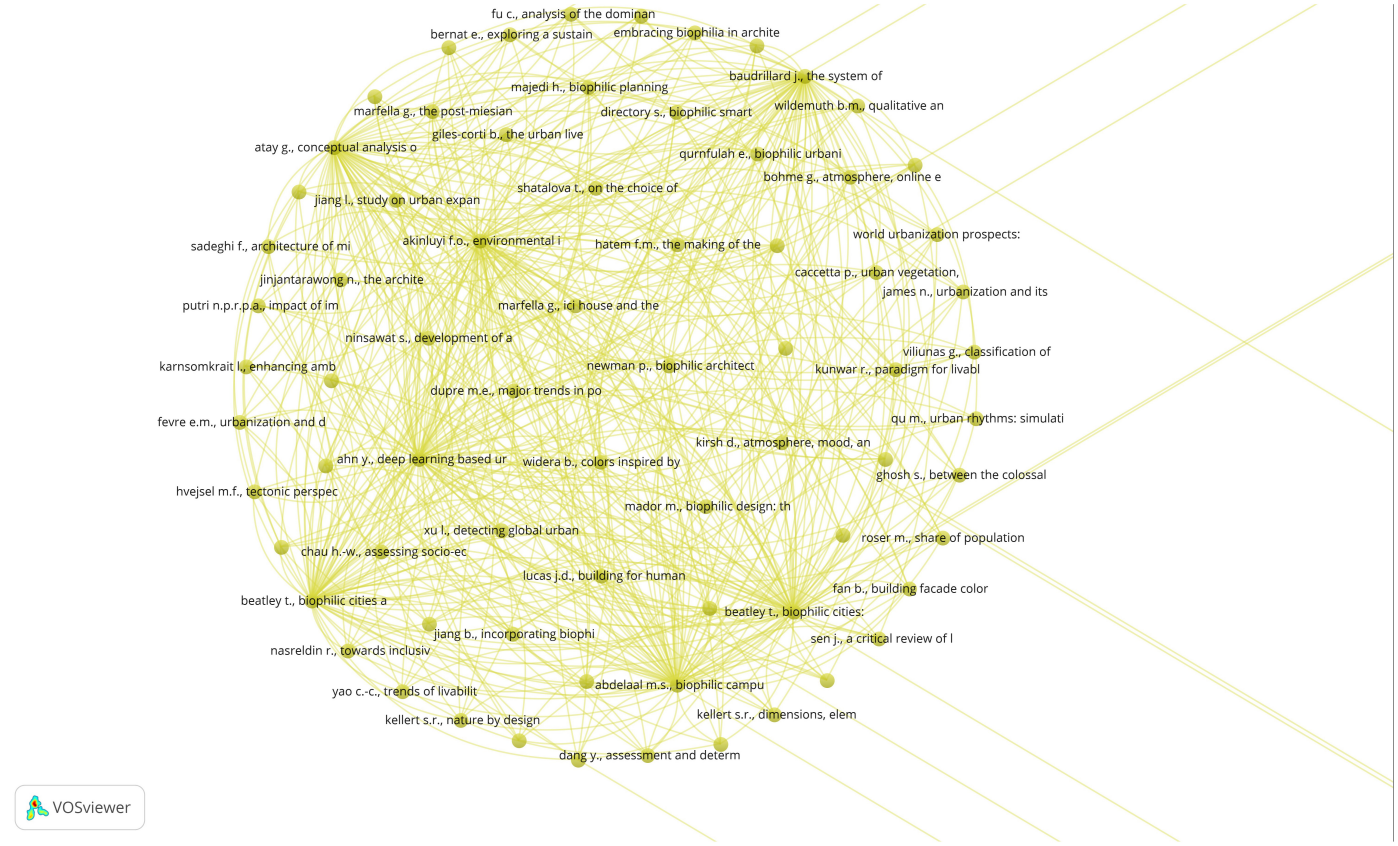
Co-citation cluster reflecting biophilic urbanism and city-scale planning literature. Source: VOSviewer / Authors Analysis (2026).

The first cluster centers on biophilic urbanism and city-scale planning (Figure 8), anchored by Beatley's foundational work on biophilic cities, alongside literature on biophilic smart cities, urban vegetation, and global urbanization trends. The presence of Kellert's core biophilic design texts at the periphery of this cluster suggests that city-scale applications of biophilic design draw directly on the field's foundational design principles, extending them from the building-scale to the scale of urban planning and policy.

**Figure 9:**
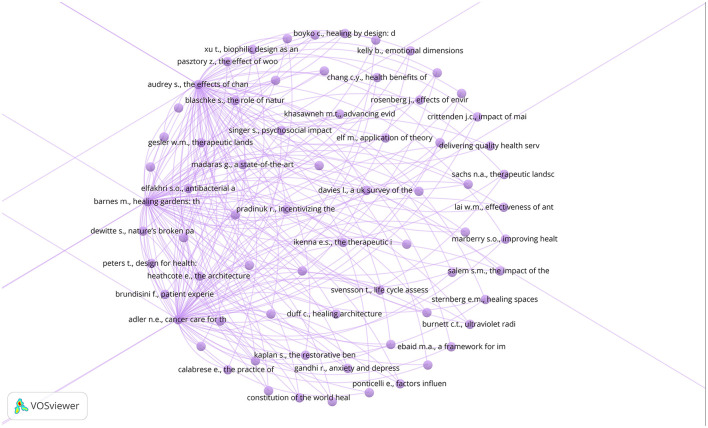
Co-citation cluster reflecting evidence-based healthcare design and restorative environments literature. Source: VOSviewer / Authors Analysis (2026).

The second cluster reflects evidence-based healthcare design and restorative environments (Figure 9), dominated by literature on healing gardens, therapeutic landscapes, and cancer care environments, alongside Kaplan's work on the restorative benefits of nature and studies addressing anxiety, depression, and patient experience. This cluster's density and internal cohesion suggest that healthcare applications constitute a well-established, heavily cross-referenced research community operating with a distinct citation base from the other two clusters.

**Figure 10:**
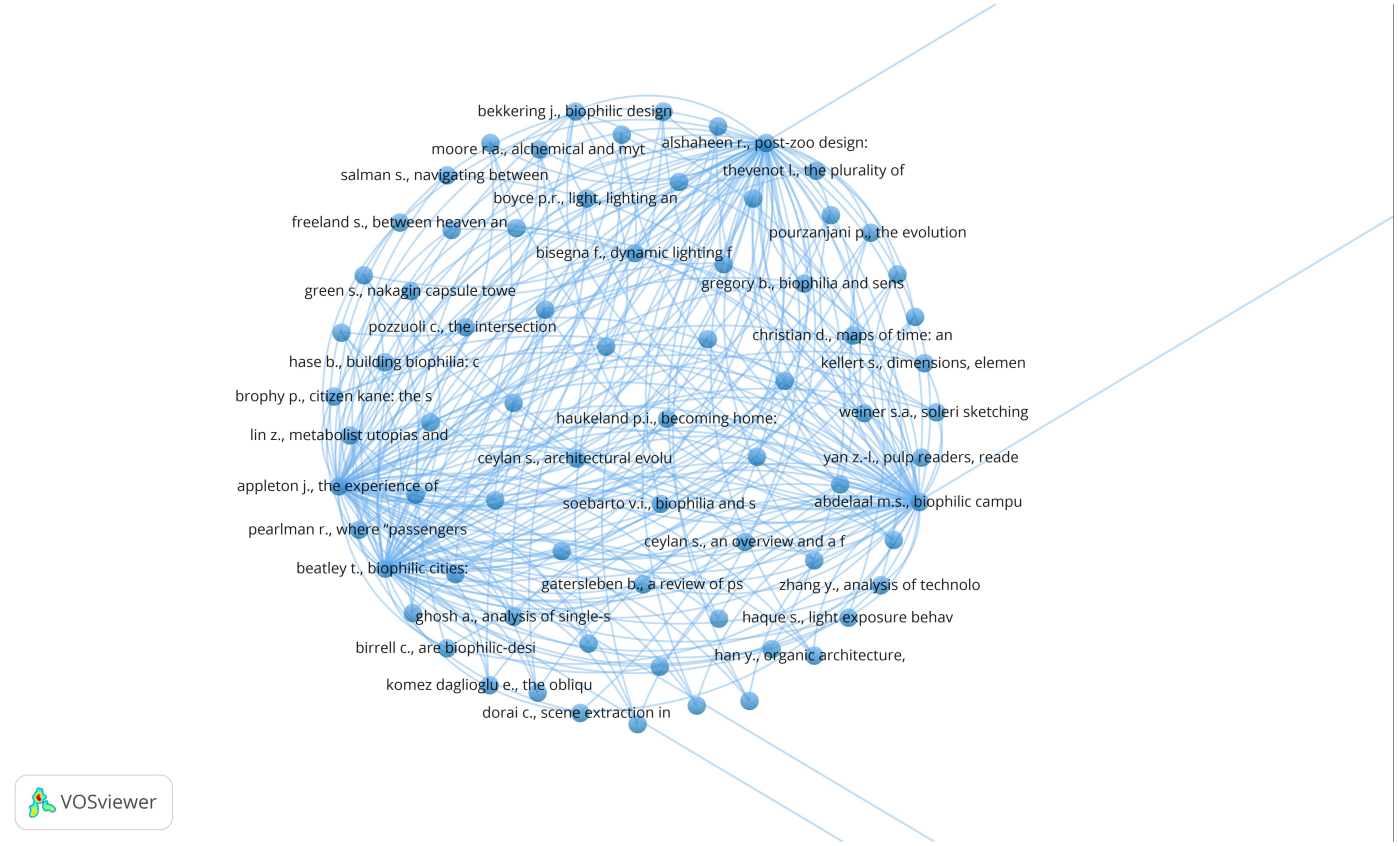
Co-citation cluster reflecting theoretical and perceptual foundations of biophilic design. Source: VOSviewer / Authors Analysis (2026).

The third cluster represents the field's theoretical and perceptual foundations (Figure 10), combining Appleton's prospect-refuge theory of landscape experience, literature on the Metabolism architectural movement and arcology, studies of lighting design and light exposure, and works addressing biophilia and human perception more broadly. This cluster is comparatively more eclectic than the other two, suggesting it functions as a shared conceptual and historical foundation from which the more applied urbanism and healthcare literatures have since diverged.

Notably, several works, including Beatley's biophilic cities scholarship and Abdelaal's work on biophilic campus design, appear as bridging references connecting multiple clusters, indicating that certain contributions serve as conceptual through-lines linking the field's theoretical, urban, and applied research strands.

Taken together, these co-citation patterns reveal an intellectual structure not visible in the keyword co-occurrence analysis alone (Section 3.3): rather than a single unified literature, biophilic architecture research rests on three largely distinct citation communities, theoretical and perceptual foundations, urban-scale planning, and healthcare-specific applications, that converge thematically but draw on substantially different underlying evidence bases.

### 3.3 RQ3: What are the dominant thematic clusters, semantic relationships, and chronological keyword evolutions that define the intellectual framework of biophilic architecture?

#### 3.3.1 Theme A: sustainable architecture, biophilic design, and urban climate resilience (red cluster)

**Figure 11:**
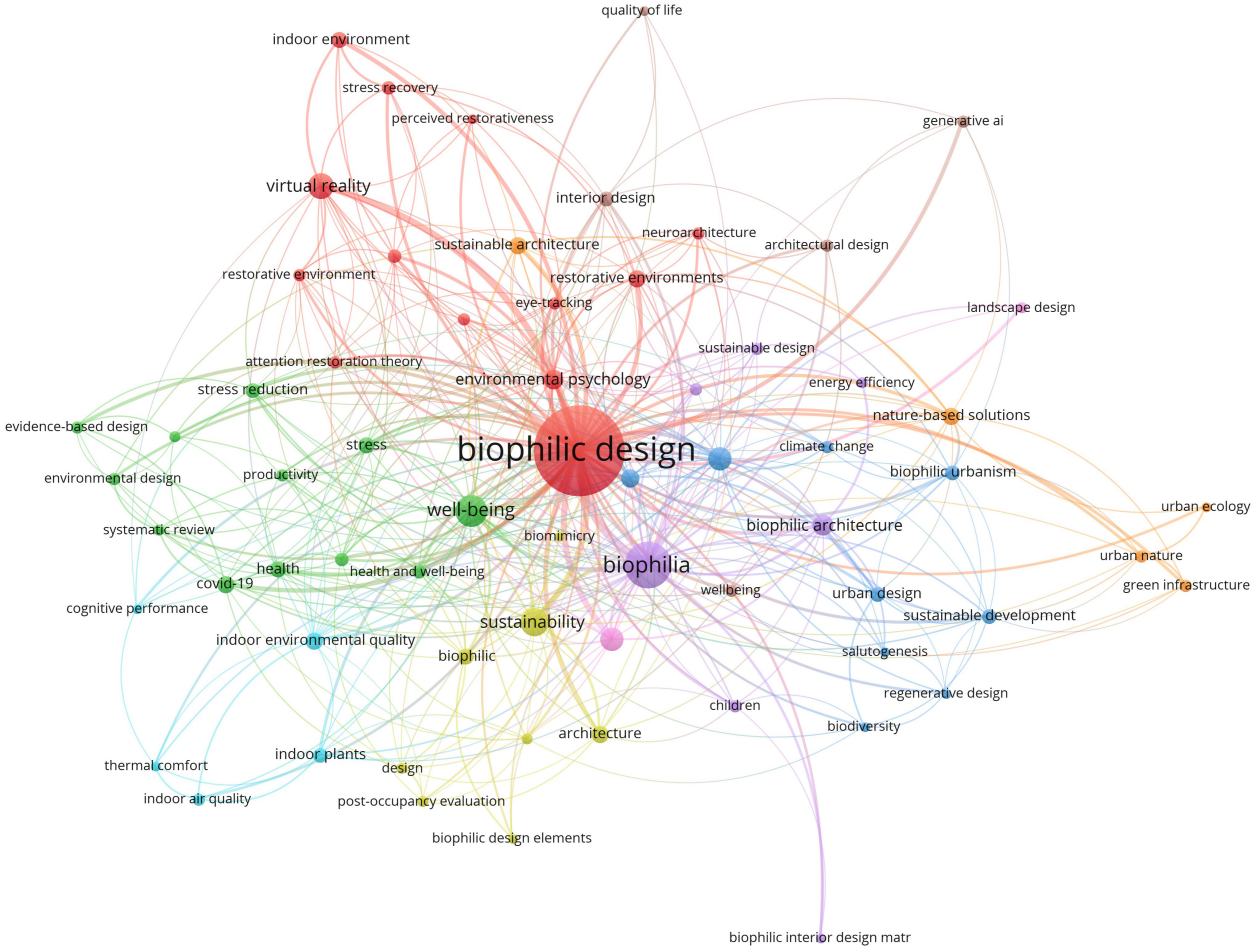
Thematic mapping of author keyword co-occurrences defining the intellectual framework of biophilic architectural research. Source: VOSviewer / Authors Analysis (2026).

The red cluster occupies the largest conceptual footprint within the co-occurrence network, serving as the foundational base that connects architectural design philosophy to broader global environmental concerns. As shown in Figure 11, this cluster is anchored by the high density macro nodes of “biophilic design,” “architecture,” and “sustainability.” Radiating outward from this core are peripheral nodes including “climate change,” “biodiversity,” “green infrastructure,” and “sustainable development.” The density of linkages across these terms demonstrates that contemporary research positions biophilic architecture well beyond the realm of isolated aesthetic preference. Rather, the literature frames it as a critical structural mechanism for urban climate adaptation, ecological preservation, and resilient urban planning.

#### 3.3.2 Theme B: indoor environmental quality and building performance physics (Green Cluster)

The green cluster centers on the technical and performance based dimensions of biophilic design within indoor environments. The major keywords in this cluster (Figure 11) include “indoor environmental quality,” “daylighting,” “thermal comfort,” and “energy efficiency.” The close connections between these terms point to a strong research interest in understanding how natural elements can improve both building performance and occupant comfort. Studies within this theme examine how features such as natural ventilation, daylight access, and passive environmental controls can reduce energy consumption while enhancing indoor comfort and overall environmental quality. The cluster as a whole reflects a growing shift toward evidence-based and performance oriented approaches to biophilic design.

#### 3.3.3 Theme C: environmental psychology, wellness, and restorative environments (blue cluster)

The blue cluster highlights the human centered dimension of biophilic architecture, drawing together keywords such as “wellbeing,” “mental health,” “stress,” “restorative environments,” and “environmental psychology” (Figure 11). This cluster reflects the growing scholarly focus on the psychological and emotional effects of the built environment on its users. The findings suggest that recent studies are moving beyond subjective accounts of human comfort, increasingly treating health and wellness as measurable outcomes within architectural design. Biophilic principles are therefore now widely explored as practical tools for reducing stress, promoting mental restoration, and enhancing the overall wellbeing of building occupants.

#### 3.3.4 Theme D: typological sector deployments and workplace productivity (yellow cluster)

The yellow cluster examines the application of biophilic design across specific building sectors, with a particular focus on workplaces, offices, educational facilities, and healthcare environments. Frequently appearing keywords include “office,” “workplace,” and “productivity” (Figure 11), pointing to growing research interest in the relationship between natural design features and human performance.

This cluster indicates that a significant body of studies now addresses the practical and economic benefits of biophilic environments, among them improved concentration, reduced stress, lower absenteeism, and increased workplace productivity. The findings also suggest that biophilic design is becoming increasingly relevant in institutional and commercial settings, where occupant performance and wellbeing are central considerations for clients and designers alike.

#### 3.3.5 Chronological evolution and mapping shifts over time

The chronological analysis of author keywords reveals a clear and progressive shift in the focus of biophilic architecture research over time. Between 2006 and 2016, most studies concentrated on conceptual discussions and theoretical explanations of biophilia, sustainability, and the relationship between humans and nature. During this period, the literature was largely shaped by the foundational contributions of scholars such as Stephen Kellert and William Browning.

**Figure 12:**
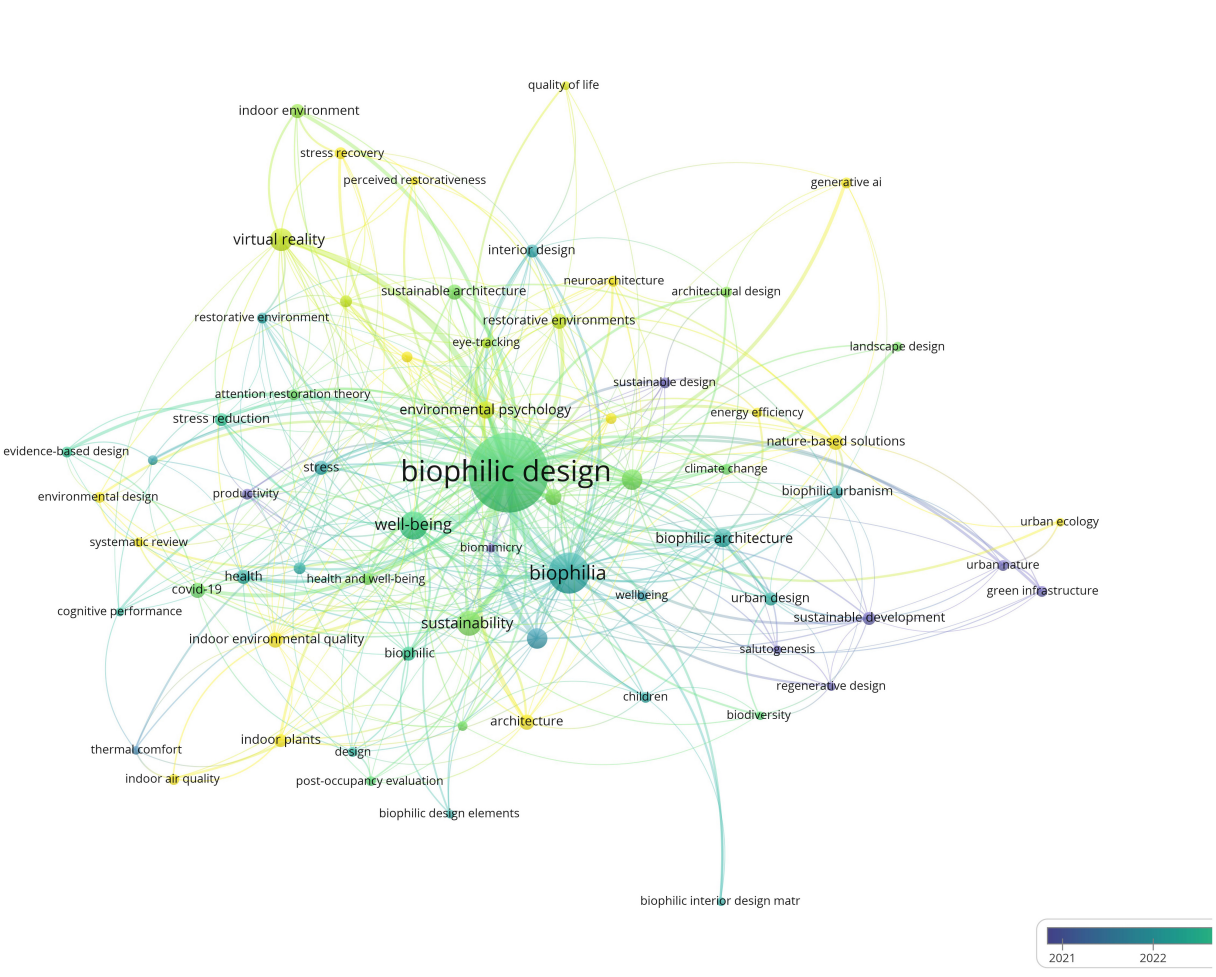
Chronological timeline overlay visualisation of keyword evolutions in biophilic design research. Source: VOSviewer / Authors Analysis (2026).

From 2017 to 2021, research attention began to move toward technical applications and building performance. As shown in Figure 12, keywords such as “green walls,” “indoor air quality,” and “simulated nature” grew more prominent, reflecting a rising interest in integrating measurable biophilic strategies into everyday architectural practice.

More recently, between 2022and 2026, the field has turned its attention toward resilience, health protection, and digital innovation. Emerging keywords such as “therapeutic architecture,” “post pandemic wellness,” “resilience,” and “virtual reality simulation” indicate that researchers are now exploring how biophilic environments can support human wellbeing in the face of climate challenges, urban stress, and global health pressures. This recent trend also points to the growing role of digital technologies and simulation tools in evaluating the effectiveness of biophilic design strategies.

**Figure 13:**
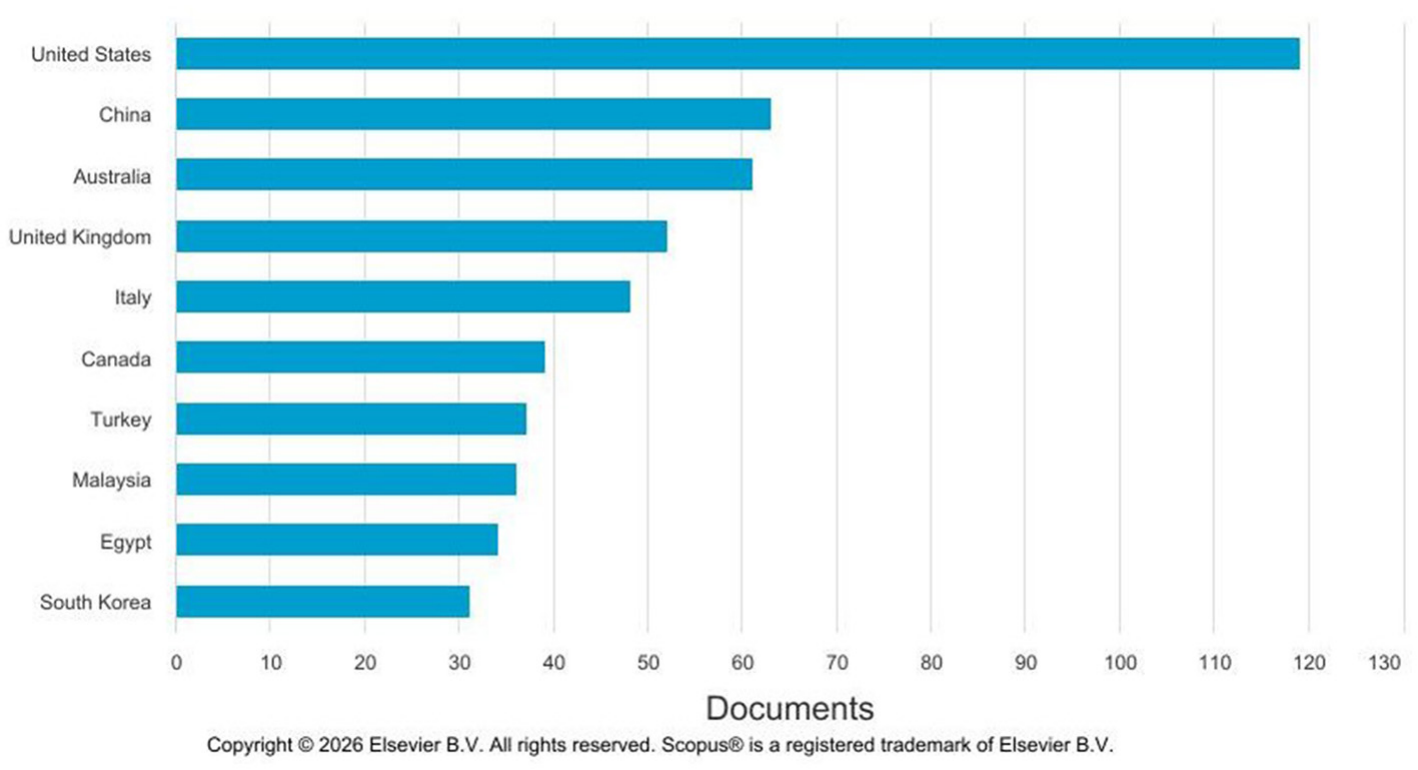
Geographic distribution of scientific output across the top 10 leading countries in biophilic design literature. Source: Scopus / Authors Analysis (2026).

### 3.4 RQ4: What critical knowledge gaps persist within the existing literature, and what strategic directions should govern future research frameworks in biophilic design?

Cross-examining the relational performance metrics and semantic keyword networks reveals three notable gaps that persist in the existing biophilic architecture literature:

#### 3.4.1 Geographic and climatic homogeneity

The findings reveal that research on biophilic design remains largely concentrated in developed countries, particularly the United States, alongside parts of East Asia and Australia (Figure 13). This concentration may partly reflect differences in research funding and database indexing rather than differences in relevance across regions, but it does suggest that existing design approaches and evaluation frameworks have so far been developed and tested predominantly under temperate climate conditions. Research focusing on hot-humid tropical regions, arid environments, and rapidly urbanizing areas across the Global South remains comparatively limited. This points to a need for more context specific studies that explore how biophilic principles can be adapted to diverse climatic and cultural conditions, drawing on locally available materials and passive design strategies.

#### 3.4.2 Socio-economic stratification and affordability gaps

The thematic analysis also reveals that practical applications of biophilic design remain heavily concentrated in commercial offices, corporate buildings, and other high budget developments. By comparison, relatively little attention has been given to affordable housing, public infrastructure, and underserved communities. This represents a gap in the literature, particularly in terms of understanding how low cost and adaptable biophilic strategies might be applied in the environments where social and environmental pressures tend to be most acute.

#### 3.4.3 Long-term longitudinal and post-occupancy validation

While a considerable number of studies have documented the short term psychological and environmental benefits of biophilic spaces, evidence on their long term performance remains limited. Few studies have undertaken extended post-occupancy evaluations that examine building efficiency, maintenance demands, or the sustained effects of biophilic environments on occupant health and wellbeing. This points to a need for more longitudinal research to determine whether the intended benefits of biophilic design hold up over time.

### 3.5 Discussions

The findings of this bibliometric analysis suggest that biophilic architectural design has progressed from a relatively niche area of research into an established multidisciplinary field that integrates architecture, environmental science, public health, and sustainability. This progression is consistent with the observations of Zhong et al. (2022), who noted the growing recognition of biophilic design as an effective strategy for improving human wellbeing while advancing sustainable development. The marked increase in publications after 2021 is consistent with the argument presented in the introduction that global concerns surrounding climate change, urbanization, and public health may have contributed to accelerating scholarly interest in nature-integrated design. The surge in research following the COVID-19 pandemic also aligns with the findings of Bahador and Mahmoudi Zarandi (2023), who argued that the pandemic highlighted the importance of healthier indoor environments and renewed attention toward nature based architectural solutions.

The thematic structure identified through the keyword co occurrence analysis reflects many of the core issues highlighted in the introduction. The prominence of sustainability, climate change, biodiversity, and green infrastructure within Theme A suggests that contemporary research increasingly positions biophilic design as more than an aesthetic design philosophy, associating it more closely with environmental resilience and sustainable urban development. This finding is in line with previous studies by Agboola (2024), Ailyn (2025), and Wijesooriya et al. (2023), which emphasized the contribution of biophilic principles to sustainable building performance and environmental quality. The strong connections between architecture and sustainability observed in the present study further suggest that researchers increasingly treat biophilic design as a component of climate responsive architectural practice.

The emergence of indoor environmental quality, daylighting, thermal comfort, and energy efficiency as dominant keywords within Theme B reinforces the shift from conceptual discussions toward measurable building performance. Earlier studies introduced biophilic design primarily as a means of reconnecting occupants with nature through visual and sensory experiences (Kellert and Calabrese, 2015; Browning and Ryan, 2020). The present findings suggest that current research increasingly evaluates biophilic interventions using objective performance indicators such as energy consumption, indoor environmental quality, and occupant comfort. This is consistent with the work of (Asojo and Hazazi 2025), (Deshpande et al. 2024), and (Tabassum and Park 2024), who demonstrated that integrating natural ventilation, daylight, and passive environmental strategies can simultaneously improve building performance and user satisfaction. Overall, the findings suggest that biophilic design is evolving from a descriptive design philosophy toward an evidence-based approach supported by measurable environmental outcomes.

The prominence of wellbeing, mental health, restorative environments, and environmental psychology within Theme C also reflects the theoretical foundations presented in the introduction. Biophilia was originally conceptualized as the innate human tendency to connect with nature (Wilson, 1984; Kellert et al., 2008), and numerous studies have demonstrated the psychological benefits associated with natural environments (Hartig et al., 2014; Richardson and Butler, 2022). The present analysis suggests that these concepts continue to shape contemporary research, with increasing emphasis on measurable health outcomes rather than solely theoretical explanations. This finding aligns with Hähn et al. (2021), Alam (2023), and Khairoowala et al. (2025), who reported that exposure to biophilic environments contributes to stress reduction, improved cognitive performance, and enhanced psychological wellbeing. The growing prominence of these keywords may therefore reflect a broader shift from conceptual discussion toward empirical evaluation of human health outcomes.

The findings relating to Theme D further reflect the applications of biophilic design across different building typologies discussed in the introduction. Previous studies have reported positive impacts of biophilic interventions in healthcare facilities, educational environments, workplaces, residential developments, and hospitality settings (Arriazu Ramos et al., 2024; Ghaziani et al., 2021; Permana et al., 2020; Ariyawansa and Perera, 2022). The strong representation of workplace, office, productivity, healthcare, and education related keywords within the present study suggests that these sectors continue to feature prominently in practical applications of biophilic architecture. This suggests that researchers increasingly recognize the economic, social, and health benefits associated with nature-integrated environments, particularly where occupant wellbeing and productivity are important design objectives.

One of the principal motivations for this study was the fragmented nature of existing biophilic literature, as identified in the introduction. The bibliometric findings are consistent with this observation, revealing multiple interconnected research domains that span sustainability, environmental psychology, building science, urban resilience, and sector specific applications. Although these research areas are strongly connected through the concept of biophilic design, they appear to develop along relatively independent disciplinary pathways. This fragmentation points to the value of bibliometric studies in providing an integrated view of the intellectual structure of the field while identifying opportunities for greater interdisciplinary collaboration.

The geographical distribution of publications also points to a persistent imbalance in global research activity. The dominance of the United States, China, and Australia indicates that most empirical evidence continues to originate from developed economies. This pattern may reflect a combination of factors, including research funding availability, database indexing practices, and language coverage, rather than a lack of relevance of biophilic design to other regions. Even so, it suggests that many existing design frameworks have so far been developed and validated primarily within temperate climatic conditions and comparatively affluent urban contexts. This observation is consistent with the concerns expressed by Zhong et al. (2022), Wijesooriya et al. (2023), and Sultan Qurraie and Bayram (2025), who emphasized the limited availability of context specific studies from developing regions. The relatively limited contribution from Sub Saharan Africa and other parts of the Global South suggests that additional research is needed to examine how biophilic principles can be adapted to different climatic, cultural, and socioeconomic conditions using locally available materials and indigenous design knowledge.

The chronological evolution of author keywords further illustrates the changing priorities within biophilic architectural research. Earlier studies concentrated largely on establishing the theoretical relationship between humans and nature through concepts introduced by Kellert and Browning. More recent publications have shifted toward resilience, therapeutic environments, digital simulation, and post pandemic wellbeing. This transition is consistent with a broader evolution of architectural research toward evidence-based design supported by digital technologies and quantitative performance evaluation. The emergence of virtual reality simulation and therapeutic architecture suggests that future research may increasingly integrate computational tools with biophilic design principles to evaluate environmental performance and user experience before construction.

Overall, the findings suggest that biophilic architectural research has undergone substantial intellectual development during the past two decades. The field appears to have expanded beyond its original theoretical foundations to encompass sustainability, building performance, environmental psychology, and practical applications across multiple building sectors. These findings are consistent with the arguments presented in the introduction and suggest that biophilic design is becoming an important research domain for informing healthier, more sustainable, and more resilient built environments. At the same time, the identified geographical and thematic gaps point to opportunities for future research, particularly within developing countries and underrepresented climatic contexts.

## 4 Conclusions and recommendations

### 4.1 Conclusion

This study presented a bibliometric analysis of global research on biophilic architectural design, drawing on publications from the Scopus database spanning 2006 to 2026. Through an examination of high frequency author keywords, the study identified major publication trends, leading contributors, and the broader intellectual structure of the field. Four major research themes emerged from the analysis: urban climate resilience, building performance and environmental control, human wellness and environmental psychology, and sector specific applications of biophilic design.

The findings demonstrate that research in biophilic architecture has evolved considerably over the period studied. Earlier work was primarily concerned with theoretical discussions of the relationship between humans and nature, whereas more recent studies place greater emphasis on measurable outcomes such as energy efficiency, occupant wellbeing, climate adaptation, and computational design approaches. This shift indicates that biophilic design is no longer viewed solely as an aesthetic or conceptual pursuit, but as a meaningful strategy for creating healthier and more sustainable built environments.

#### 4.2.1 Limitations

This study has several limitations. First, it draws exclusively on Scopus, which excludes relevant work indexed only in other databases such as Web of Science or regional, non-English repositories. Second, the search was limited to English language publications, which likely contributes to the geographic concentration of results in the United States, Australia, and East Asia, and should be considered when interpreting the reported Global North to Global South imbalance. Third, only journal articles and review papers were included, excluding conference papers and other gray literature common in architecture and design research. Fourth, the keyword co-occurrence analysis depends on author-assigned terms, so some thematic boundaries reflect variation in author language as much as genuine conceptual divisions. Finally, this study is primarily descriptive. Although it incorporates co-authorship, citation, and co-citation analyses, it does not include advanced citation impact analyses such as citation burst detection, bibliographic coupling, or field-normalized citation metrics. Furthermore, the study does not include a qualitative content analysis of individual publications. Future research should integrate these approaches to provide a more comprehensive understanding of the intellectual development, research influence, and knowledge structure of biophilic architectural research.

#### 4.2.2 Implications for architectural practice

The study underscores the need for architects and design professionals to embrace more practical and performance based biophilic strategies. Rather than relying on decorative elements such as indoor plants alone, biophilic principles should be embedded from the earliest stages of the design process, drawing on tools such as BIM modeling, daylight analysis, and natural ventilation planning. This integrated approach has the potential to improve indoor environmental quality while simultaneously reducing energy consumption.

Equally important is the need to extend the reach of biophilic design beyond commercial buildings and high end developments. Architects and planners should work toward developing affordable and adaptable biophilic solutions for public housing, schools, and healthcare facilities, particularly in areas where occupants are already contending with elevated levels of environmental and social stress.

#### 4.2.3 Recommendations for future research

Future research should give greater attention to regions that remain underrepresented in the current literature, particularly hot-humid, arid, and sub Saharan environments. Studies in these contexts should explore the potential of local materials, indigenous knowledge, and vernacular design traditions in advancing biophilic architecture in ways that are culturally grounded and climatically appropriate. There is also a pressing need for research on affordable and inclusive biophilic design approaches that extend the benefits of the field to low income communities, rather than concentrating them in premium developments.

Beyond geographical and social inclusivity, future researchers should prioritize long term post-occupancy evaluations to develop a more robust understanding of how biophilic environments perform over time, and what lasting effects they have on building efficiency, occupant health, and operational costs.
